# Exploration of the integration of microbiology and immunology emerging topics into undergraduate medical education

**DOI:** 10.1080/10872981.2024.2336331

**Published:** 2024-04-05

**Authors:** Margaret E. Bauer, Samina Akbar, Timothy J. Bauler, Jessica Chacon, Erin E. McClelland, Shawn Staudaher, Yuan Zhao

**Affiliations:** a Microbiology and Immunology, Indiana University School of Medicine, Indianapolis, IN, USA; b Biosciences Division, Marian University College of Osteopathic Medicine, Indianapolis, IN, USA; c Homer Stryker M.D. School of Medicine, Western Michigan University, Kalamazoo, MI, USA; d Medical Education, Texas Tech University Health Sciences Center El Paso, Lubbock, TX, USA; e Educational Affairs, Sam Houston State University College of Osteopathic Medicine, Conroe, TX, USA; f Molecular and Cellular Biology, Sam Houston State University College of Osteopathic Medicine, Conroe, TX, USA

**Keywords:** Emerging topics, microbiology and immunology, undergraduate medical curriculum, integration of basic science, clerkship readiness

## Abstract

**Purpose:**

Medical school educators face challenges determining which new and emerging topics to incorporate into medical school curricula, and how to do so. A study was conducted to gain a better understanding of the integration of emerging topics related to microbiology and immunology in the undergraduate medical curriculum (UME).

**Methods:**

An anonymous survey with 17 questions was emailed to medical school faculty who teach immunology and/or microbiology through the DR-Ed listserv, the American Society for Microbiology (ASM) Connect listserv, and attendees of the Association of Medical School Microbiology and Immunology Chairs (AMSMIC) Educational Strategies Workshop. Participants were asked about experiences, perceptions, and the decision-making process regarding integrating emerging topics into UME.

**Results:**

The top emerging topics that were added to the curriculum or considered for addition in the last 10 years included COVID-19, Zika virus, mRNA vaccines, and Mpox (formerly known as monkeypox). Most respondents reported lectures and active learning as the major methods for topic delivery, with most faculty indicating that formative assessment was the best way to assess emerging topics. Content experts and course directors were the most cited individuals making these decisions. Top reasons for incorporating emerging topics into curricula included preparing students for clinical treatment of cases, followed by demonstrating the importance of basic science, and opportunities to integrate basic science into other disciplines. Challenges for incorporating these topics included making room in an already crowded curriculum, followed by content overload for students.

**Conclusions:**

This study describes the rationale for integrating emerging topics related to microbiology and immunology into UME, and identifies the current new and emerging topics, as well as the main methods of integration and assessment. These results may be used by medical educators to inform curricular decisions at their institutions. Future studies will include developing innovative learning modules that overcome barriers to integration.

## Introduction

Modern preclinical medical curricula are increasingly integrated, as integration is firmly established as being beneficial to learning [1–3]. Horizontal integration in preclinical medical curricula has led to replacement of traditional discipline-based courses by organ system-based courses and other innovative curricula [1]. Accordingly, ‘the preclinical curriculum’ is an intricate puzzle with many pieces, all of which must efficiently work together under severe time and resource constraints to deliver the optimal content for medical students. Medical educators who are part of the preclinical curriculum team of scientists and clinicians that teach medical students are continuously challenged to determine the depth of science medical students need to know. Some scientific disciplines required by medical students, including microbiology and immunology, are constantly changing, for example as infectious organisms emerge or evolve and novel immune-based treatments are approved based upon new scientific knowledge.

With limited curricular time, medical school microbiology and immunology educators may struggle to determine when and how to incorporate new and emerging topics into the curriculum. This is complicated by the fact that many of these topics are taught in courses where they are not the course director or lead. ‘New and emerging’ (and re-emerging) topics were defined for this study as content that has not traditionally been taught or emphasized, but due to scientific/medical advances, increased numbers of patient cases, and/or increased public awareness of the topic, should be considered for addition to the preclinical medical curriculum. Consideration of emerging topics, such as COVID-19 and novel Food and Drug Administration-approved cancer immunotherapeutics, is required of microbiology and immunology educators more than some other basic science disciplines, as the fields of infectious diseases and applied immunotherapies change over time. Determining which emerging topics should be added to the preclinical medical curriculum is a careful balancing act for microbiology and immunology educators. ‘New’ content is particularly relevant and exciting for a medical learner, and all stakeholders want physicians to have the most up-to-date scientific and medical knowledge. However, the rate of acquisition of new medical and scientific knowledge means that including ‘everything’ into the curriculum is impossible, as curricula are already overcrowded [4].

Consensus learning objectives for microbiology and immunology were published in 2009, which were intended to define the minimal content medical students needed for competency [5]. The frequency of medically-relevant changes within the fields of microbiology and immunology since 2009 demonstrates that relying on consensus documents to determine whether a new topic should be added to the curriculum is impractical. Thus, there is no established method by which faculty may determine how to integrate new emerging microbiology or immunology content. The only published article about emerging microbiology topics described the frequency at which applied microbiology content, such as hospital infection control, antimicrobial stewardship, and global health were incorporated into curricula of allopathic medical schools in 2016 [6]. No published studies have investigated emerging topics in immunology in preclinical medical curricula.

The aims of this study were to determine which factors are considered by microbiology and immunology educators in the United States before they incorporate a new and emerging topic into their curriculum, define the microbiology and immunology topics considered new and emerging at a single snapshot in time (2022), and explore best practices of how these topics are being integrated. These results will be useful for educators to consider when incorporating emerging topics in their discipline-specific portions of the premedical curriculum at their respective institutions.

## Methods

Exempt status for the research project was granted by the Institutional Review Board of each investigator’s institution (Western Michigan University IRB: WMed-2022–0962, Marian University IRB: IRB S22.172, Indiana University Human Research Protection Program: 17130, Sam Houston State University IRB: IRB-2022-321, Texas Tech University: E23034).

A 17-question anonymous survey (Appendix I) was constructed using Qualtrics (Provo, UT) and emailed to faculty who teach immunology and/or microbiology at allopathic and osteopathic medical schools using the DR-Ed listserv, the American Society for Microbiology (ASM) Connect listserv, and the attendee list of the 2022 Association of Medical School Microbiology and Immunology Chairs (AMSMIC) Education Strategies Workshop. The survey asked participants about experiences, perceptions, and the decision-making process regarding integrating emerging topics into Undergraduate Medical Education (UME). Demographic data on the type and size of medical school was also collected for analysis. A total of 69 participants agreed to respond to the survey, with 40 giving informed consent and completing all questions (not including two open-ended questions). Our analysis was confined to the 40 complete responses from U.S. medical schools.

The survey question types fell into three categories: multiple choice, ranking, and a slider scale. For the ranking questions, respondents ranked a set of choices. In the slider scale question, respondents moved a slider to choose between 1 (lowest) and 5 (highest). For a complete list of survey questions see Appendix I.

The type of question determined the statistical analysis used for each question. Chi-square tests were used to determine if there was a significant difference between allopathic and osteopathic respondents. For multiple selection multiple choice questions, contingency tables were generated to examine conditional relationships between the choices (i.e., how often one choice occurred in the presence of another choice). A *t*-test was used to determine if there was a significant difference between the two choices in the slider scale question where participants were asked to grade the importance of public perception and clinical impact in the decision to include an emerging topic in the curriculum. Inter-rater reliability for the ranking questions was measured using Kendall’s *W* [7] and statistical significance was measured using a follow-up Chi-square test. Data processing, statistical computations, and visualizations were performed with Python 3.11 using the pandas, scipy, and seaborn libraries. Narrative responses (*n* = 37) from the open-end question ‘how do you define an emerging topic in microbiology and immunology’ were analyzed using constant comparison analysis [8] and classical content analysis [9]. Qualitative statistics were computed using Dedoose 9.0.90.

## Results

The 40 respondents of the study were from 31 medical schools across the United States. Among the 31 schools, 19 (61%) were allopathic medical schools and 12 (39%) were osteopathic medical schools. Relatively more osteopathic schools were represented in this study compared to the current U.S. totals of 155 (80%) allopathic and 38 (20%) osteopathic schools. The participating institutions have a variety of different campus environments, with an average incoming class size ranging from 49—550 per campus, the number of campuses ranging from 1—9, and the ratio of microbiology and immunology faculty to campuses ranging from 0.8:1 to 20:1. Among the 40 respondents, 15 (37.5%) report teaching microbiology only, 10 (25%) teach immunology only, and 15 (37.5%) teach both disciplines.

To test for differences in responses to the multiple choice and single-selection questions between faculty at allopathic and faculty at osteopathic medical schools, chi-square tests were conducted, and no significant differences were identified for any of the questions (α=0.05).

### Emerging topics for UME curriculum

Medical school educators were asked to provide their definition for an emerging topic in microbiology and immunology. Qualitative analysis of the responses showed the most common code was *new topic and knowledge*. Among 15 responses that were mapped to this code, nine specifically mentioned new or re-emerging infectious diseases or organisms. Three responses mentioned immunology related topics. The second most common code was *impact on patient health and clinical practice*; nine responses were mapped to this code. The third common code was *increasing clinical incidence;* six responses were mapped to this code. Other less common codes included *not on licensing exam yet* and *recently or not yet in the textbook*. To summarize, the respondents defined emerging topics in microbiology and immunology as a new topic or knowledge related to new and emerging infectious diseases and new applications of immunology that have impacted patient health and medical practice with increasing clinical incidence.

**Figure 1.**
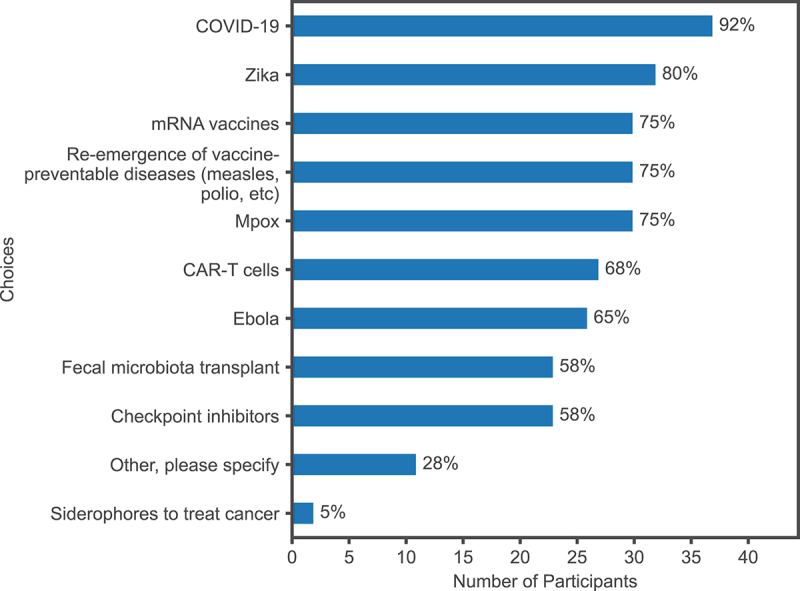
Selected emerging topics added or considered for addition to the undergraduate medical education curriculum, *N* = 40.

Next, microbiology and immunology educators were provided with a list of emerging topics and asked which of these have been added or considered for addition to the curriculum in the last 10 years. The list was developed by the authors, who are content experts of microbiology and immunology, and consulted colleagues (five) in the field. Survey respondents selected between 2—11 topics (*M* = 6.8, SD = 2.5); with the exception of siderophores to treat cancer, each topic was selected by at least 20 (50%) of the respondents (Figure 1). The popularity of the selected topics align with our perception of the prevalence of recent media coverage. Some educators also wrote in their own topics, but none of these were noted by more than one respondent.

### Reasons for incorporating emerging topics into the UME curriculum

Participants were asked to separately rank medically relevant and public perception factors that suggest an emerging topic warrants inclusion in the curriculum. Agreement amongst respondents was found to be statistically significant for both the medically relevant factors (Kendall’s *W* = 0.52, χ^2^ (4, *N* = 40) = 83, *p* < 0.001) and public perception factors (Kendall’s *W* = 0.57, χ^2^ (5, *N* = 40) = 114, *p* < 0.001). Among medically relevant factors, significant number of cases/deaths worldwide and regionally/locally were ranked as the top two choices, respectively (Table 1). Newly-approved treatments and newly-changed management guidelines were ranked as less relevant clinical factors for including new and emerging topics into the curriculum. Among the public perception factors, national and local news coverage were ranked as the top two reasons, respectively, for an emerging topic to be considered for addition to the curriculum. Social media posts and commercials for treatments or vaccines were the third and fourth ranked public perception reasons for curricular addition, respectively, with family inquiries as the least relevant rationale (Table 2).

**Table 1.** Ranking of clinical impact or important triggers in suggesting an emerging topic warrants inclusion in undergraduate medical education curriculum (mean was calculated as the average ranking for each choice, 1 is the top choice), *N* = 40.

	Rank					
Choice	1	2	3	4	5	Mean
Significant number of cases/deaths worldwide	24	10	4	2	0	1.6
Significant number of cases/deaths seen in region/local community	6	18	7	9	0	2.48
Newly approved treatment	5	6	21	7	1	2.83
Newly changed management guidelines	2	6	8	20	4	3.45
*Other-Open ended response	3	0	0	2	35	4.65

*Top ranked open-ended responses included: issues that affect healthcare delivery, mass media coverage, and illustrating important principles.

**Table 2.** Ranking of public perception or popular media coverage in suggesting an emerging topic warrants inclusion in undergraduate medical education curriculum (mean was calculated as the average ranking for each choice, 1 is the top choice), *N* = 40.

	Rank						
Choice	1	2	3	4	5	6	Mean
It’s on the national news nightly	31	6	2	1	0	0	1.32
Local news stations are covering the topic	1	19	11	6	3	0	2.77
See regular social media posts	0	10	10	9	10	1	3.55
See commercials for new treatment or vaccine	4	2	9	14	9	2	3.7
Family members ask you about it	1	3	8	9	18	1	4.08
*Other-Open ended response	3	0	0	1	0	36	5.58

*Top ranked open-ended responses included: covered in prominent medical journals, important in the clinical media, students have not indicated much specific interest.

**Figure 2.**
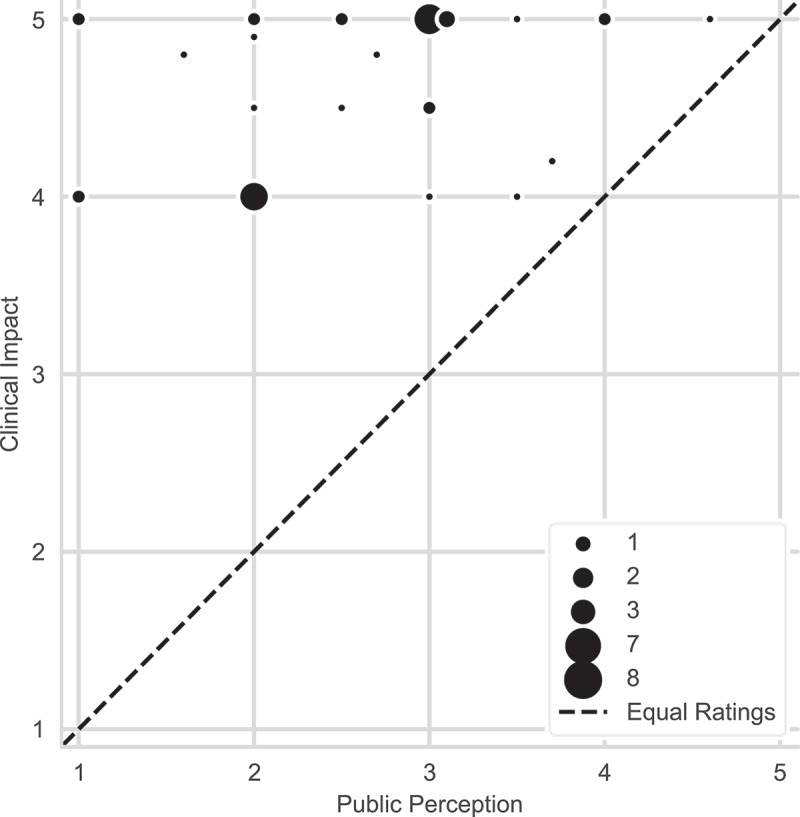
Ratings of clinical impact versus public perception in the decision to include an emerging topic in the curriculum. Due to overlapping data, the size of each point indicates the number of respondents who selected each score pair. The diagonal dashed line represents an equal rating for clinical impact and public perception with points above the line indicating that the participant rated clinical impact higher than public perception, *N* = 40. (survey question 10 was used for this data).

To compare the relative importance of public opinion vs clinical impact in the decision to include an emerging topic into medical curricula, participants were asked to use sliding scales (from 1-least important to 5-most important). Clinical impact (*M* = 4.64, SD = 0.44) was rated as significantly more important than public opinion (*M* = 2.59, SD = 0.85) by survey respondents with a two-tailed paired *t*-test result of *t*(39) = 13.5, *p* < 0.001. Notably, every participant rated clinical impact higher than public perception (Figure 2).

Medical educators were also asked to rank the educational rationale for discussing new microbiology and immunology content in UME. Agreement amongst respondents was found to be statistically significant (Kendall’s *W* = 0.52, χ^2^ (4, *N* = 40) = 83, *p* < 0.001). The top-ranked reason for incorporating emerging topics into the curriculum was preparing students for treating cases in the clinic, followed by seizing additional opportunities to demonstrate the importance of basic science, and to integrate basic science into other disciplines (Table 3). Satisfying student interest in a topic and preparing students for standardized medical exams were deemed less important, and using the emerging topic to add additional basic science content to the curriculum was ranked least important of the options provided.

**Table 3.** Ranking of reasons for incorporating emerging topics into curriculum (mean was calculated as the average ranking for each choice, 1 is the top choice), *N* = 40.

	Rank							
Choice	1	2	3	4	5	6	7	Mean
Prepare students for seeing cases in the clinic	24	7	2	3	4	0	0	1.90
Integrate basic science into other disciplines (like ethics of cost of treatment, vaccine hesitancy, public health, etc)	9	7	11	5	5	3	0	2.98
Demonstrate the importance of basic science	3	14	12	5	5	1	0	2.95
Satisfy student interest in the topic	1	6	4	10	11	8	0	4.20
Prepare students for standardized/board exams	2	6	4	7	6	14	1	4.38
Allows more basic science to be taught	0	0	6	10	9	14	1	4.85
*Other-Open ended response	1	0	1	0	0	0	38	6.75

*Top ranked open-ended responses included: help students understand and contextualize a topic in the media.

### Decision making process for integrating emerging topics in the UME curriculum

Respondents were also asked to select who is involved in deciding if an emerging topic should be incorporated into the curriculum; 36 (90%) selected content experts and 32 (80%) selected course directors, whereas only five (12.5%) selected curriculum committee, two (5%) selected associate dean of education or similar role, and two (5%) selected department chair. Additionally, open-ended responses included case writing team, discipline team, and committee specific for reviewing and approving pre-clerkship learning objectives, with each selected by a single respondent. Content experts and course directors were not only selected most often, 29 (72.5%) respondents selected both. Also, all five respondents that indicated a role for the curriculum committee also selected content expert and course director. Similarly, both respondents that selected associate dean of education selected content expert and course director. Together, this suggests that the decision to include emerging topics is most often made at the content expert and course director level, and when higher levels of leadership are involved, content experts and course directors remain involved in the decision-making process.

### Teaching methods and assessments for emerging topics

We next asked several questions about which teaching modalities and assessment strategies are used when adding emerging topics to the curriculum. First, respondents were asked to select the single best teaching modality for emerging topics. The most common choice was lecture with 18 (45%) respondents, then active learning methods (case-based learning and discussion) with 12 (30%), team-based learning (TBL) with 4 (10%), problem-based learning (PBL) with 2 (5%), asynchronous modules with 2 (5%), and other with 2 (5%). While lecture was the top choice, when all three active learning choices are combined, there is a tie with 18 (45%) for lecture and 18 (45%) choosing one of the three active learning choices. Interestingly, of the active learning methods, case-based and discussion were chosen far more frequently than TBL and PBL. For the best method of assessment, formative assessment was chosen by 18 (45%) respondents, summative examinations by 10 (25%), low-stake summative (quizzes) by 5 (12.5%), and no assessment by 4 (10%). Multiple free-text responses also commented that the teaching and assessment methods should depend on the new and emerging topic itself, and the level of depth required to adequately cover the topic (data not shown).

### Challenges for integrating emerging topics in the UME curriculum

Medical educators were also asked to rank the obstacles and challenges that may make integrating emerging topics in the UME curriculum a challenge. Agreement amongst the respondents for this question was statistically significant (Kendall’s *W* = 0.72, χ^2^ (5, *N* = 40) = 143, *p* < 0.001). Making room in an already full course or curriculum was the leading challenge identified by respondents, with concerns about content overload for students the second most important challenge identified (Table 4). Concerns about lack of assessment on national examinations, faculty time-constraints for new content development, and administrative concerns were deemed less important barriers.

**Table 4.** Challenges for incorporating emerging topics in the curriculum (mean was calculated as the average ranking for each choice, 1 is the top choice), *N* = 40.

	Rank						
Choice	1	2	3	4	5	6	Mean
Making room in an already full course or curriculum	29	4	5	1	1	0	1.52
Content overload for students	6	18	12	4	0	0	2.35
Content not covered yet on board exams	4	10	6	11	9	0	3.27
Time constraints for faculty	1	7	14	15	3	0	3.30
Curriculum committee has to approve all such changes	0	1	3	8	27	1	4.60
*Other-Open ended response	0	0	0	1	0	39	5.95

Open ended responses included: student resistance due to new/emerging topics not covered by board exam and faculty being unfamiliar with new topics.

## Discussion

How and when to incorporate new and emerging topics into undergraduate medical school curricula is a challenge frequently faced by medical school faculty. While the COVID-19 pandemic made microbiology and immunology faculty particularly aware of the need to regularly reevaluate and update their content, there have been many other significant infectious disease outbreaks and immune-based therapeutics that have become important in the past decade. In this study, microbiology and immunology educators from medical schools across the country were surveyed to define these new and emerging topics, evaluate what factors they consider before incorporating a new and emerging topic into their curricula, how this content should be taught and assessed, and what challenges must be overcome.

Efforts to integrate emerging topics in other disciplines have been addressed in previous studies. For example, in 2022, after talking to various stakeholders, Sullivan *et al*. conducted a comprehensive analysis to determine where and how to insert climate change and health into their curriculum [10]. They devised a 6-step model based on Thomas *et al* to achieve horizontal and vertical integration of climate change and health into the curriculum [11]. Other groups have conducted multi-institution surveys to determine what emerging topics should be taught.

One study published in 2013 surveyed medical schools in the United Kingdom and Ireland on teaching of biological weapons and bioterrorism [12]. Of the 34 medical schools that answered their survey, only 6 (17.7%) had specific teaching on biological weapons and bioterrorism in the curricula, but most schools did not. From free responses, the reasons for why this topic was not part of the curriculum included that the teaching schedule was too busy, or the topic was not compulsory. In addition, some regarded this field of study as a postgraduate subject that was not appropriate for undergraduates, and it would be very rare for a junior doctor to see these types of cases.

All 40 medical educators that participated in this study have included or considered including emerging topics in their curriculum, demonstrating that faculty recognize the importance of teaching emerging topics. There is some correlation between the prevalence and recency of an infectious disease or immune-based therapy and the percentage of respondents who indicated they had considered the topic for integration. For example, COVID-19 was the most selected topic (*n* = 37, 92%); given its recent global significance, this result was not surprising. Somewhat less frequently identified was Zika virus (*n* = 32, 80%), a pathogen whose expanded geographic distribution during the 2014–2015 epidemic revealed a previously unappreciated effect on fetal development [13]. Similarly, mRNA vaccines (*n* = 30, 72%) were ranked higher than Chimeric Antigen Receptor (CAR)-T cells (*n* = 27, 68%), as mRNA vaccines were broadly distributed worldwide during the COVID-19 pandemic, while CAR-T cells, FDA-approved in 2017, are an effective cancer therapy targeted for a narrower population [14]. Given that survey respondents were all medical educators, the finding that the 1—5 rating of clinical significance was higher (and statistically significant), than public perception in determining which emerging topics to add to the UME curricula, was unsurprising. However, respondents still ranked public perception or media coverage 2.59 on the 1—5 scale, suggesting that indicators of social impact remain important contributing factors in this decision. For example, some topics of lesser clinical significance may be included to address student interests or concerns arising from non-medical sources of information.

Respondents to our study suggested lectures (*n* = 18, 45%), case-based learning, and discussions (*n* = 12, 30%) as the best methods for teaching emerging topics. It was interesting that TBL/PBL were chosen less frequently. This may be because educators find it easier to add a slide or case to an existing lecture than create an entirely new TBL/PBL. Additionally, because these are new and emerging topics, there may be insufficient verified clinically relevant information to create a new TBL/PBL. With the trend of UME moving toward integrative models and the adaptation of virtual learning due to the recent pandemic, some additional ideas for integrating emerging topics include introducing them through asynchronous learning modules, small case studies, or TBL activities in system-based courses. Several studies have reported innovative ways to teach individual emerging topics. Chiu *et al*. share their experience in developing and implementing a student-led virtual COVID-19 course, while Kemp et al. describe a collaborative self-directed learning COVID-19 elective [15,16]. In another study, Kabelitz *et al*. report on the formation of an Education Committee of the International Union of Immunological Societies (IUIS). This committee administers three to four one-week courses per year, that focus on the most relevant topics and health issues facing specific countries or regions around the world [17]. The optimal curriculum design and teaching methods for emerging topics likely vary based on the impact of the disease or immunotherapeutic and the depth of the scientific literature on the topic. For example, for COVID-19 it is reasonable to explore the opportunity in the curriculum to have multiple, focused active learning sessions, as well as develop the connection between various concepts and disciplines, such as public health and ethics. Some other emerging topics, such as immune checkpoint inhibitors, may merit simply replacing a small fraction of an existing lecture on cancer immunotherapies due to a reduced worldwide impact.

Although a new or emerging topic may be important and exciting to scientists, and faculty may desire to devote hours of teaching to the topic, it is not feasible for every program to identify space in the curriculum to offer an independent session or course for emerging topics. As UME curricula prepare future physicians for lifelong learning and promote the culture of self-identification, including in the curriculum all content that might be relevant to physicians in future decades should not be the goal of medical educators. In fact, based on the survey, the biggest challenge for incorporating emerging topics in UME is making room in an already full course and curriculum. The concerns of curriculum overload for UME has lasted over a century [4,18–20]. Several solutions have been proposed to address the issue and a few studies report the successful implementation of a less comprehensive curriculum, which usually involves a huge multidisciplinary team effort [21–23]. When deliberate curriculum planning cannot take place, purposeful and creative integration of new/emerging topics in curriculum may require removing older, ‘classic’ topics with less medical relevance from the curriculum entirely. Content experts and course directors are the major decision makers regarding what and how to incorporate new topics in the curriculum, per our survey data. In the future, faculty development and encouragement of scholarly design and dissemination of education materials and/or modules for these new topics will be very beneficial for the medical education community and may alleviate the burden of content development.

This study was subject to several limitations. First, there were strong selection effects as the survey was not distributed randomly. A medical education conference attendee list and two education-focused listservs were used to distribute the survey. While there was diversity in the sample, methods to correct selection effects, such as post-stratification, were not used due to a limited sample size. Also, faculty who attend educational conferences and subscribe to medical education-focused email list servs likely have distinct viewpoints compared to faculty who do not. In addition, several respondents came from the same institution, and respondents may teach only microbiology, only immunology, or both. There are also limitations for the rank-based questions. Differences between ranks are subjective for each respondent, making absolute comparisons between ranked items impossible. For instance, a respondent may rank their first choice nearly equal to their second choice, or they could consider their first choice far above their second; a ranking question cannot distinguish between these two possibilities. Due to these limitations, this study was not designed to test a specific hypothesis, but instead its purpose was to describe how education-focused faculty incorporate and teach emerging topics in microbiology and immunology.

Another limitation is this study defined the ‘new and emerging’ microbiology and immunology topics at a specific moment in time, Fall 2022. Accordingly, COVID-19 was the most-frequently selected topic. While the study also identified Mpox as a highly-relevant new topic, barring a recurrence of an Mpox outbreak five years from now, Mpox would likely not be included in the curriculum of 75% of respondents in 2027. This limitation also demonstrates the importance of faculty defining the process of how and when new and emerging topics are added to (or removed from) the curriculum, because the medically-relevant microbiology and immunology topics are regularly changing.

The core curriculum of the UME should ensure the competency of medical graduates to deal with the common or important clinical problems that they are likely to encounter in future clinical practice [24]. As we reflect on the COVID-19 pandemic that has deeply impacted all aspects of medical school education, we conclude that it is important to include emerging topics in the medical school curriculum to prepare students for changes in clinical practice and the needs of future doctors.

## Data Availability

Complete survey data used/analyzed in this study are available from the corresponding author on request.

## Appendix I Survey Questionnaire

(1) Please answer the following demographic questions regarding the school you are affiliated with.
  a. Name of the school
  b. Average incoming class size/year
  c. Number of Campuses
  d. Number of faculty who primarily teach microbiology/immunology content (all campuses combined)
(2) Do you primarily teach Microbiology, Immunology, or both?
  - Microbiology
  - Immunology
  - Both
(3) How is the pre-clerkship microbiology and immunology taught? (Select all that apply)
  - Stand-alone microbiology course or stand-alone immunology course
  - Combined microbiology and immunology course
  - Part of foundational sciences course
  - Combined with other disciplines (e.g., Hematology, etc.)
  - Integrated into organ systems course
  - Part of fully integrated curriculum (no separate micro/immuno course)
(4) What major modalities are used to teach the pre-clerkship microbiology/immunology content? (Select all that apply)
  - Lectures (including active learning components as part of an overall lecture)
  - Asynchronous modules
  - Team-based learning (TBL)
  - Problem-based learning (PBL)
  - Other active learning – Case-based learning, discussions, etc
  (5) How do you define an emerging topic in microbiology and immunology? [open response]
  (6) Please identify emerging topics you added to your curriculum or considered adding to your curriculum in the last 10 years. (Select all that apply)
  - COVID-19
  - Monkeypox
  - Zika
  - Ebola
  - Re-emergence of vaccine-preventable diseases (measles, polio, etc)
  - mRNA vaccines
  - CAR-T cells
  - Checkpoint inhibitors
  - Fecal microbiota transplant
  - Siderophores to treat cancer
  - Others [open response]
(7) Who is involved in the decision to incorporate an emerging topic into the curriculum? (Select all that apply)
  - Content expert
  - Course director
  - Department chair
  - Curriculum Committee
  - Associate Dean of Education or similar administrative entity
  - Others [open response]
(8) What levels of clinical impact or important triggers suggest an emerging topic warrants inclusion in the curriculum? (Please rank order)
  - Newly-approved treatment
  - Newly-changed management guidelines
  - Significant number of cases/deaths worldwide
  - Significant number of cases/deaths seen in region/local community
  - Others [open response]
(9) What level of public perception or popular media coverage suggests an emerging topic warrants inclusion in the curriculum? (Please rank order)
  - See regular social media posts
  - It’s on the national news nightly
  - Family members ask you about it
  - Local news stations are covering the topic
  - See commercials for new treatment or vaccine
  - Others [open response]
(10) On a sliding scale, please indicate your opinion of the relative importance of public opinion vs clinical impact in the decision to include an emerging topic into your curriculum. (5-likert scale, 1-least important, 5-most important)
  - Clinical impact
  - Public perception or popular media coverage
(11) What is the major modality that you think would be best to teach these emerging topics?
  - Lectures (including active learning components as part of an overall lecture)
  - Asynchronous modules
  - Team-based learning (TBL)
  - Problem-based learning (PBL)
  - Other active learning – Case-based learning, discussions, etc
  - Other [open response]
(12) What is the best way to assess these newly emerging topics?
  - No assessment
  - Formative only
  - Low-stakes summative (quizzes)
  - Summative examinations
  - Others [open response]
(13) If you add a major emerging topic into the curriculum, does something else have to be removed? If yes, who is involved in the decision of what topic to remove? [select all that apply]
  - Content expert
  - Course director
  - Department chair
  - Curriculum Committee
  - Associate Dean of Education or similar administrative entity
  - Others [open response]
  - Nothing needs to be removed
(14) Rank the reasons for incorporating emerging topics into the curriculum.
  - Demonstrate the importance of basic science
  - Allows more basic science to be taught
  - Integrate basic science into other disciplines (like ethics of cost of treatment, vaccine hesitancy, public health, etc)
  - Satisfy student interest in the topic
  - Prepare students for seeing cases in the clinic
  - Prepare students for standardized/board exams
  - Others [open response]
(15) Rank the obstacles/challenges that work against adding emerging topics into the curriculum.
  - Making room in an already full course or curriculum
  - Curriculum committee has to approve all such changes
  - Time constraints for faculty
  - Content not covered yet on board exams
  - Content overload for students
  - Other: Please specify
(16) What are the resources you use to develop the session with emerging topics? (Select all that apply)
  - Textbook
  - Journal articles
  - CDC guidelines
  - UptoDate or similar resources
  - Others: Please specify
(17) Is there anything else you want to tell us about integrating emerging microbiology and immunology topics into medical curricula, pre-clerkship or clerkship?
